# Ketamine Boluses Are Associated with a Reduction in Intracranial Pressure and an Increase in Cerebral Perfusion Pressure: A Retrospective Observational Study of Patients with Severe Traumatic Brain Injury

**DOI:** 10.1155/2022/3834165

**Published:** 2022-05-21

**Authors:** Bradley A. Dengler, Oliver Karam, Colleen A. Barthol, Aaron Chance, Laura E. Snider, Clare M. Mundy, Michael T. Bounajem, William C. Johnson, Moustafa M. Maita, Paola M. Mendez-Gomez, Ali Seifi, Shaheryar Hafeez

**Affiliations:** ^1^Department of Neurosurgery, Walter Reed National Military Medical Center, Bethesda, MD, USA; ^2^Division of Pediatric Critical Care Medicine, Children's Hospital of Richmond at VCU, Richmond, VA, USA; ^3^Clinical Pharmacy Specialist, Neuroscience Intensive Care Unit, Department of Pharmacotherapy & Pharmacy Services, University Health System, San Antonio, TX, USA; ^4^Department of Neurosurgery, University of Texas Health Science Center San Antonio, San Antonio, TX, USA; ^5^Department of Neurology, University of Alabama Birmingham, Birmingham, AL, USA; ^6^Family Medicine Health Training Program, San Diego, CA, USA; ^7^Department of Neurosurgery, University of Utah, Salt Lake City, UT, USA; ^8^Department of Anesthesiology, University of Texas Health Science Center at San Antonio, San Antonio, TX, USA; ^9^Department of Neurology, University of Texas Health Science Center at San Antonio, San Antonio, TX, USA

## Abstract

**Background:**

Increased intracranial pressure (ICP) and hypotension have long been shown to lead to worse outcomes in the severe traumatic brain injury (TBI) population. Adequate sedation is a fundamental principle in TBI care, and ketamine is an attractive option for sedation since it does not commonly cause systemic hypotension, whereas most other sedative medications do. We evaluated the effects of ketamine boluses on both ICP and cerebral perfusion pressure (CPP) in patients with severe TBI and refractory ICP.

**Methods:**

We conducted a retrospective review of all patients admitted to the neurointensive care unit at a single tertiary referral center who had a severe traumatic brain injury with indwelling intracranial pressure monitors. We identified those patients with refractory intracranial pressure who received boluses of ketamine. We defined refractory as any sustained ICP greater than 20 mmHg after the patient was adequately sedated, serum Na was at goal, and CO_2_ was maintained between 35 and 40 mmHg. The primary outcome was a reduction in ICP with a subsequent increase in CPP.

**Results:**

The patient cohort consisted of 44 patients with a median age of 30 years and a median presenting Glasgow Coma Scale (GCS) of 5. The median reduction in ICP after administration of a ketamine bolus was −3.5 mmHg (IQR −9 to +1), and the postketamine ICP was significantly different from baseline (*p* < 0.001). Ketamine boluses led to an increase in CPP by 2 mmHg (IQR −5 to +12), which was also significantly different from baseline (*p* < 0.001).

**Conclusion:**

In this single-institution study of patients with severe traumatic brain injury, ketamine boluses were associated with a reduction in ICP and an increase in CPP. This was a retrospective review of 43 patients and is therefore limited in nature, but further randomized controlled trials should be performed to confirm the findings.

## 1. Introduction

Severe traumatic brain injury (TBI) continues to plague the worldwide medical community with over 5.48 million cases each year, which accounts for over half of all trauma-related deaths [1]. The initial clinical care and subsequent intensive care unit (ICU) care can substantially impact the morbidity and mortality of these patients. Periods of hypotension along with raised intracranial pressure (ICP) have been linked to increased mortality and worse outcomes in patients with severe TBI [2–6]. The 2016 Brain Trauma Foundation guidelines recommend treating ICP >22 mmHg and maintaining systolic blood pressure (SBP) >90 mmHg [7].

Most recent TBI studies along with expert opinion divide treatment algorithms for increased ICP into three tiers of therapy. The first tier involves using short-acting analgesic and sedative agents such as fentanyl, propofol, and midazolam to increase sedation [8–10]. These sedatives can cause hypotension, which can lead to worse outcomes in this patient population [11, 12]. The ideal sedative would, therefore, provide blood pressure support while at the same time decrease ICP.

Ketamine is a dissociative anesthetic that binds to the N-methyl-d-aspartate (NMDA) receptor, leading to a blockade of excitatory synaptic activity. The use of ketamine in brain injury has previously been reported to be a contraindication due to early studies showing an increase in ICP following its administration [13, 14]. These studies were largely reported in children with obstructive hydrocephalus, and recent data suggest ketamine might actually be beneficial in these patients [15]. In addition, ketamine does not lead to vasodilation or induce hypotension, as seen with most other anesthetics and sedatives. In contrast, it can lead to increased levels of norepinephrine in the blood and induce an overall sympathomimetic response [16]. For these reasons, ketamine becomes an attractive choice for sedation in severe TBI.

The objective of this study was to determine the effects of bolus doses of ketamine on both ICP and cerebral perfusion pressure (CPP) in a patient population with severe TBI.

## 2. Materials and Methods

### 2.1. Patient Population

This is a retrospective observational study in which patients admitted to a tertiary university-affiliated adult neurocritical care unit in San Antonio, Texas, USA, were identified between 1 January 2014 and 31 December 2017. Patients were identified by searching the pharmacy charge database and determining which patients received intravenous (IV) ketamine. The list of patients who had received ketamine was then evaluated, and only patients who had sustained a severe TBI, had an ICP monitor in place, and were on mechanical ventilation were selected for inclusion. This list was further refined by including only patients with refractory ICP who were already receiving osmotherapy, adequately sedated, and being administered analgesia infusions. Patients were cared for at the discretion of the attending neurosurgeon and neurointensivist. Institutional review board approval was obtained, waiving the requirement for written informed consent as this was a retrospective chart review.

All data were collected prospectively in the medical record up to hospital discharge, interinstitutional transfer, or death, whichever occurred first, and then evaluated retrospectively. Data collected on admission included age, weight, gender, presenting Glasgow Coma Scale (GCS), intracranial lesions, epidural hematoma (EDH), subarachnoid hemorrhage (SAH), subdural hemorrhage (SDH), contusions and axonal injury, and acute ischemic stroke (AIS). Data collected during the ICU course included the type of ICP monitor (external ventricular drain (EVD) and/or Codman monitor (Integra LifeSciences, NJ, USA). Additional data collected included bolus and/or continuous infusion doses of ketamine, fentanyl, propofol, dexmedetomidine, benzodiazepines, pentobarbital, and paralytics, along with hypertonic saline and mannitol boluses. The ICP was recorded at the beginning of each hour, and the ketamine boluses were administered during that hour.

### 2.2. Intervention

The intervention of interest was all IV boluses of ketamine administered by the treating team. Ketamine infusions for sedation/analgesia and bolus dosing of ketamine were given at the discretion of the attending neurointensivist. Although no official protocol was in place at the time of the study, it was agreed upon practice of the neurointensivists at the time that ketamine was given when ICP was sustained greater than 20 mmHg and documented during that hour, serum sodium was between 150 and 155, if an EVD was in place it was open and draining, and other sedative boluses had failed to improve the ICP; therefore, ketamine boluses were only given if all other therapies failed to improve the ICP. In addition, ketamine boluses were considered if there was concern that boluses of other common sedatives (propofol, versed, and fentanyl) would cause significantly more hypotension.

### 2.3. Cointerventions

Patients admitted with severe TBI were managed according to the 3rd edition of the Brain Trauma Foundation guidelines which were the published guidelines during the duration of the study. ICP was maintained <20 mmHg, and SBP was maintained >90 mmHg [17, 18]. All patients were intubated and sedated with invasive ICP monitoring in place [19]. All patients received a hypertonic saline by either continuous infusion of 3% NaCl or intermittent boluses of 6.4%, 3% NaCl, or 23.4% NaCl in order to maintain a serum sodium between 150 and 155 mmol/L. Treatment of elevated ICP was performed using a tiered approach to therapy with all patients initially treated with the head of bed elevation to 30 degrees and maintenance of carbon dioxide (CO_2_) between 35 and 40 mmHg. Transition to more aggressive therapy including sedation boluses, mannitol, decompressive hemicraniectomy, pentobarbital coma, and paralysis was all at the discretion of the attending neurosurgeon and neurointensivist. A frontotemporal-parietal craniectomy was performed for all patients who had a decompression, and extra-axial mass lesions were removed, but parenchymal lesions were not. ICP was treated if there was a sustained elevation in ICP greater than 20 mmHg for more than 5 minutes that was not attributed to coughing or routine nursing care and documented in the last hour by the nursing staff. Boluses of ketamine were only given after ensuring all other parameters including head of bed elevation, PaCO_2_, serum sodium, and adequate sedation were ensured and never if the ICP was lower than 20 mmHg. Ketamine boluses were dosed at 2 mg/kg. Adequate sedation was defined as an RASS of −3 to −4.

### 2.4. Outcomes

The primary outcome measure was the proportion of ketamine boluses that resulted in both an improved ICP and CPP, measured on an hourly basis (as recorded in the electronic medical record). This outcome was computed a posteriori based on the presence of a decrease in ICP and a concomitant increase in CPP.

The secondary outcomes were change in ICP and CPP from the preketamine bolus baseline (in mmHg).

### 2.5. Statistical Analysis

Results are expressed as the median and interquartile range (IQR) or frequencies and proportions. Fisher's exact probability test was used to undertake unadjusted univariate tests in order to establish an association between the primary outcome and categorical variables. The Mann–Whitney *U* test was used for continuous variables. Correlations between two continuous variables were analyzed using Spearman's correlation test.

Logistic regression was used to compare odds ratios of improving both ICP and CPP, whereas forward stepwise linear regressions were used to identify factors independently associated with change in ICP and CPP, which means starting with no variables in the model, testing the addition of each individual variable and measuring the model's goodness of fit (as the adjusted *R*^2^), adding other variables whose inclusion gives the most improvement of the fit, and repeating this process until no further improvement is achieved.

Quadratic regressions with 95% confidence intervals were used to identify the correlation between baseline ICP and change in ICP and baseline CPP and change in CPP. The models were adjusted for multiple measures. We defined the efficacy zone of ketamine boluses as the zone of a decrease in ICP above a certain baseline ICP value (identified by the regression line) and an increase in CPP below a certain baseline CPP value (identified by the regression line). The groups were further classified by graphic representations according to significant variables identified in the regression model (decompressive craniectomy for ICP changes and paralytic infusion for CPP changes, respectively). We performed a subgroup analysis in which we included only the ketamine boluses that were administered as the sole intervention documented during that hour and when the ICP was greater than 20 mmHg. A *p* value <0.05 was considered significant. All tests were two-sided. All statistical analyses were performed with SPSS version 26 for Mac (SPSS, Chicago, IL, USA).

## 3. Results

There were 69 patients initially identified, and after review of those charts, 43 patients were deemed able to be included in the analysis. The remaining 26 patients did not have ketamine bolus data and were only placed on continuous ketamine infusions. Of those 43 patients, there were 216 individual boluses of ketamine administered. Of the 216 ketamine boluses, 98 (45%) still had elevated ICP greater than 20 mmHg after the bolus. Of those patients, 68 (68%) still had an overall decrease in their ICP, but the decrease was not enough to bring the ICP less than 20 mmHg. The patient cohort consisted of 32 males and 11 females with a median age of 27 years (IQR 20–40), presenting the GCS of 5 (IQR 3 to 7) and the median ketamine bolus dose of 150 mg (IQR 100 to 200) (Table 1). The majority of patients were admitted with either a cerebral contusion or a SDH.

**Figure 1:**
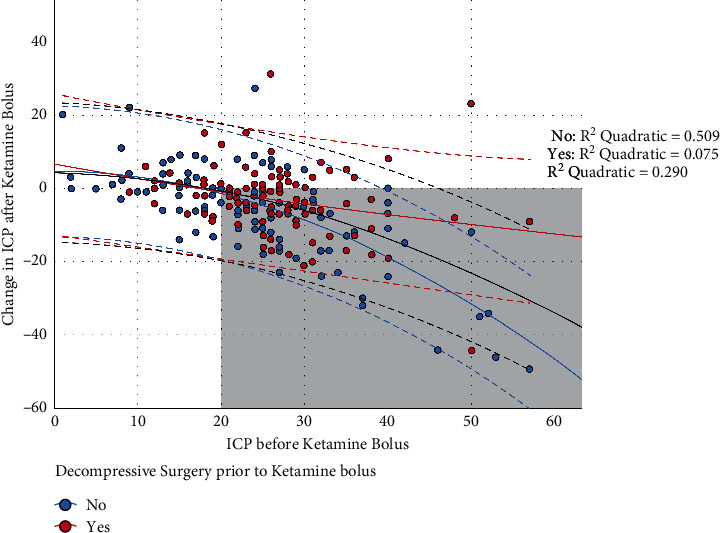
Change in ICP after ketamine bolus according to baseline ICP values. The blue circles represent patients without decompressive surgery, whereas the red circles are those with decompressive surgery. The bold lines are the regression lines (black for the whole population and red and blue for those with and without decompressive surgery, respectively), whereas the dashed lines are the 95% confidence intervals around the regression lines. The gray zone represents events where the ICP decreased the greatest according to the regression model.

**Figure 2:**
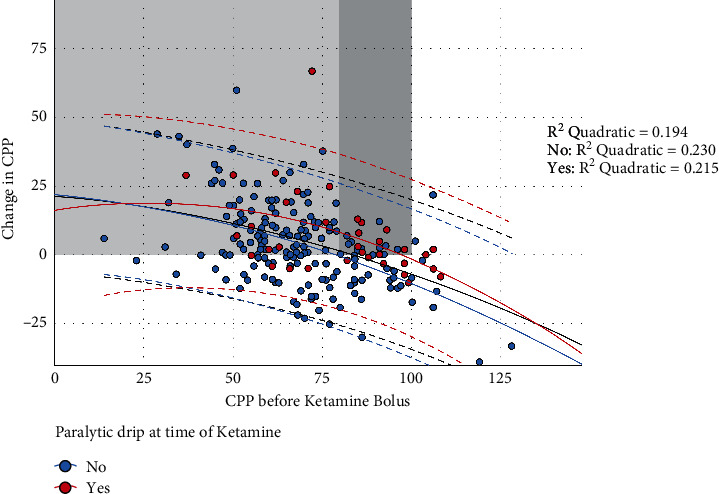
Change in CPP after ketamine bolus according to baseline CPP levels. The blue circles represent patients without paralytic drips, whereas the red circles are those with paralytic drips. The bold lines are the regression lines (black for the whole population and red and blue for those with and without paralytic drips, respectively), whereas the dashed lines are the 95% confidence intervals around the regression lines. The gray zone represents events where the CPP increased according to the regression model the greatest.

The proportion of ketamine boluses that resulted in both an improved ICP and CPP was 46% (99/216). The median change in ICP after ketamine bolus was −3.5 mmHg (IQR −9 to +1), which was a significant decrease from baseline (*p* < 0.001). The median change in CPP after ketamine bolus was an increase in 2 mmHg (IQR −5 to +12), which was a significant increase from baseline (*p* < 0.001). Univariate analysis is available in the online supplemental appendix (available here). The greatest effect on ICP was in patients with an ICP greater than 20 mmHg (Figure 1), while the greatest effect on CPP was in those patients whose CPP was less than 80 mmHg (Figure 2).

ICU length of stay and refractory elevated ICP had favorable associations with ketamine administration. ICP decreased by 1.2 mm Hg for each additional ICU day. For each mm Hg increase in ICP, there was a 0.4 mm Hg decrease in ICP after the ketamine bolus. In other words, this means that the higher the ICP and the longer the patient was in the ICU, the greater the effect ketamine bolus had on decreasing ICP. Obesity and decompressive craniectomy had a negative linear association with ketamine administration, and ICP showed an increase in these patient populations after a ketamine bolus (Table 2).

**Figure 3:**
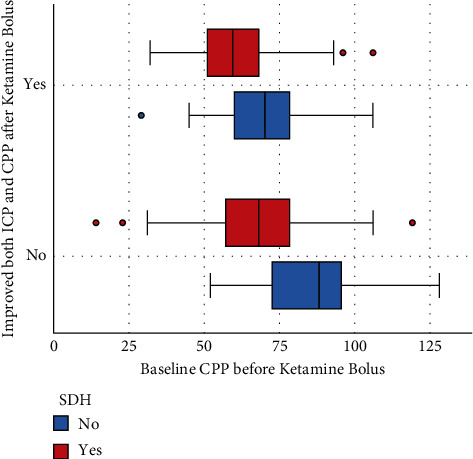
Boxplots of the baseline CPP according to the improvement of both ICP and CPP and the presence of SDH. Patients who had lower CPP and the presence of a SDH showed greatest improvement in both ICP and CPP values after a ketamine bolus.

CPP had a favorable response to ketamine administration in the presence of a paralytic infusion, concomitant benzodiazepine infusion, or hemicraniectomy. The presence of paralytics or benzodiazepines during the ketamine bolus raised the CPP by 8.7 and 7.0 mmHg, respectively (Table 2). When evaluating the effects of ketamine boluses on a decrease in ICP with a concomitant increase in CPP, the prebolus CPP (adjusted OR for 1 mmHg 0.98, 95% CI 0.96 to 0.99, and *p*=0.03) and the presence of a SDH (adjusted OR 0.4, 95% CI 0.21 to 0.77, and *p*=0.006) were independently associated with improvement in both parameters.  Figure 3 demonstrates that patients with lower CPP and a SDH showed the most benefits from administration of a ketamine bolus.

In the subgroup of patients who received only a bolus of ketamine for ICP >20 mmHg, the proportion of ketamine boluses that resulted in both an improved ICP and CPP was 49% (63/128). The median change in ICP after ketamine bolus was −5 mmHg (IQR −11 to 0, *p* < 0.001). The median change in CPP after ketamine bolus was an increase of 3 mmHg (IQR −3 to +13, *p* < 0.001). The greatest effect on CPP was on those patients whose CPP was less than 80 mmHg. The median number of boluses per patient was 4 (IQR 2 to 6).

## 4. Discussion

Ketamine is a dissociative anesthetic that works primarily as an NMDA-R antagonist but has also shown to be an effective analgesic with potential neuroprotective properties [11, 20]. It was first introduced as a dissociative anesthetic for use in neurological surgery in 1970 since it would not suppress respiratory function and had an overall sympathomimetic effect on the cardiovascular system [21]. Ketamine quickly fell out of favor in the neurosurgical and anesthesia community because of initial reports of increasing ICP in these specific patient populations. These initial studies were completed in patients who were not intubated, had intracranial mass lesions or obstructive hydrocephalus, and therefore not comparable to the severe TBI population [13, 14, 22, 23]. Recently, a systematic review evaluating the use of ketamine in TBI showed stability in ICP values with ketamine administration [16]. Three of these studies evaluated ketamine boluses (the others evaluated ketamine infusions). Two (one adult and one pediatric) showed a decrease in ICP after ketamine boluses when compared to no intervention, while one did not show a benefit of ketamine boluses over fentanyl boluses in adults [24–26]. Only one study showed an increase in CPP in a pediatric population [25]. This makes our paper one of the few to our knowledge in the literature to describe both an increase in CPP and a decrease in ICP in an adult population with severe traumatic brain injury. These studies showing a possible improved outcome effect along with ketamine's effect on the overall hemodynamic status to make it a very appealing drug for patients with severe TBI [11].

In our study, there was a subgroup of patients that showed both a decrease in ICP and an increase in CPP after administration of a ketamine bolus. This is consistent with the findings in children published by Bar-Joseph et al. which showed a 30% reduction in ICP with a subsequent increase in CPP and in the majority of studies in the systematic review [16, 25]. Our data did not show a dramatic response, but this is likely since our ICP values were measured hourly and were not recorded in the first few minutes after ketamine administration as was performed in the Bar-Joseph study. Given the quick onset and relatively short half-life of ketamine, it is possible that we would also have seen a greater improvement each minute after ketamine administration, but we still were able to demonstrate a sustained reduction in ICP [27]. In addition, the primary outcome was met in less than 50% of the ketamine boluses and overall in about half of the patients. This is concerning and speaks to the need for further studies. However, it might be due to the fact that we did not have minute-to-minute evaluation of ICP after the ketamine bolus. Therefore, it is possible there was a more dramatic, yet short-lived, effect that did not last until the next ICP recording.

The explanation for the ICP decrease after ketamine administration is still unknown, but likely related to the overall sedative effects of ketamine [11]. In addition, there is evidence that ketamine can cause both an increase and a decrease in cerebral blood flow (CBF) based on the underlying vascular reactivity [28]. One of the biggest factors determining CBF is related to changes in arterial levels of CO_2_. Patients who are not mechanically ventilated with adequately maintained CO_2_ at 35–40 mmHg might have an increase in CBF due to the slight hypoventilation induced by ketamine. This hypoventilation might lead to hypercapnia, which in turn might lead to increased ICP [13, 22, 28]. In addition, if a patient has intact cerebral autoregulation, the increase in CPP will lead to vasoconstriction and therefore a decrease in ICP [29]. Up to 85% of patients can have impaired autoregulation within three to five days after their injury [30]. The fact that there was a synergistic effect and greater reduction in ICP with coadministration of midazolam is likely related to the fact that midazolam is a sedative and decreases the cerebral metabolic rate of oxygen demand (CMRO_2_) [31].

Another interesting finding of our review is that patients with decompressive hemicraniectomy actually had a slight increase in ICP after administration of a ketamine bolus. This finding is likely explained by alteration in the pressure reactivity index caused by the cranial defect. The pressure reactivity index is defined as the ability of the cerebral vessels to respond to changes in arterial blood pressure with a reactivity index defined as a moving correlation between the mean ICP and the mean arterial pressure. A negative pressure reactivity index signifies normal working cerebral autoregulation in that an increase in the mean arterial pressure leads to a decrease in ICP due to vasoconstriction. A positive reactivity index, therefore, signifies altered cerebrovascular reactivity since an increase in mean arterial pressure will lead to an increase in ICP [32]. Multiple studies have shown that, immediately following decompressive hemicraniectomy, the pressure reactivity index is positive and therefore disturbed. This phenomenon occurred in some studies up to 72 hrs after the decompressive craniectomy [29, 33, 34]. Further evaluation of cerebral blood flow after decompressive craniectomy has shown a focal increase in CBF unilateral to the side of the decompression within the first 24 hrs after surgery. This hyperperfusion can last up to one month postoperatively [35]. It is possible that decompressive hemicraniectomy leads to dysfunctional cerebral pressure reactivity with a local hyperemia [35]. Ketamine and its sympathomimetic effects can therefore lead to increased mean arterial pressure, with subsequent increased ICP due to the positive pressure reactivity index. In addition, the hemodynamic effects of ketamine can worsen the hyperemia leading to more cerebral edema and likely higher ICP.

In the future, further prospective studies could evaluate ketamine's influence on ICP in various patient populations. Since this is one of the only medications that have shown some efficacy at both decreasing ICP and increasing CPP, it might be worthwhile in specific subgroups of patients with severe traumatic brain injury, such as those who responded appropriately to the initial bolus. Further studies comparing ketamine's overall ability to control ICP compared to ability of other commonly used sedatives in the ICU would be important. Other studies evaluating the effect of ketamine on cerebral oxygenation along with its impact on cerebral autoregulation would likely be beneficial too.

A few significant limitations exist in this study. The first is that it is a retrospective review of patients from a single institution. Second, the ICP values were measured hourly and not necessarily immediately after the ketamine bolus, so a larger response to ketamine that occurred immediately after the bolus was unable to be observed; however, it could be also possible we were not able to capture worsening ICP, the exact magnitude or the intervention being unknown. In addition, some of the ICPs appear to be in the normal range prior to the ketamine bolus. As those values were recorded hourly by the bedside nurse, it is likely there was a higher ICP spike during the hour that was not recorded by the nurse but for which ketamine was administered. Outcomes were difficult to assess as there was no control group, and confounding variables such as bolus doses of other medications could have partly contributed to the reduction in ICP, although this was controlled for in the statistical analysis. In addition, the lack of a formal protocol in the ICU for the administration of ketamine limits the generalizability of the data along with the fact that the specific RASS values, PaCO_2_ values, and Na values at the time of the bolus were not recorded. We were also not able to assess a potential interaction between the timing of the hemicraniectomy and the effect of the ketamine boluses. We did not either collect data on potential adverse events associated with ketamine boluses. Finally, we did not have data in the medical record to determine why some patients only received 1-2 boluses before stopping, but it was likely because there was no change in the ICP.

## 5. Conclusion

In this retrospective review of patients with severe TBI and refractory ICP, we showed a significant association between a ketamine bolus and a reduction in ICP and an increase in CPP. This is one of the only studies to evaluate the effect of ketamine boluses on both ICP and CPP and should lead to future hypotheses in patients with severe TBI. Randomized controlled trials are needed to better evaluate the effect of ketamine boluses and determine if there is an outcome benefit to its use as a sedative and/or rescue therapy in severe TBI patients.

## Figures and Tables

**Table 1:** Demographics, cointerventions, and outcomes according to the effect on intracranial pressure and cerebral perfusion pressure.

Variables	Whole population	Improved ICP and CPP after ketamine bolus		*P* value
		Yes (*n* = 17)	No (*n* = 26)	
Age (years)	30 (20; 44)	33 (23; 40)	26 (19; 30)	0.14
Gender (male)	77% (33/43)	74% (17/23)	80% (16/20)	0.86
Weight (kg)	78 (65; 95)	82 (68; 95)	76 (64; 99)	0.62
Presenting GCS	5 (3; 7)	4 (3; 6)	6 (3; 8)	0.76
EDH	9% (4/43)	9% (2/23)	10% (2/20)	0.76
SAH	74% (32/43)	79% (18/23)	70% (14/20)	0.3
SDH	65% (28/43)	52% (12/23)	80% (16/20)	0.13
Contusions	56% (24/43)	52% (12/23)	60% (12/20)	0.2
AIS	2% (1/43)	4% (1/23)	0% (0/20)	0.99
Type of monitoring				
EVD	40% (17/43)	35% (8/23)	45% (9/20)	0.7
Codman	37% (16/43)	39% (9/23)	35% (7/20)	
Both	23% (10/43)	26% (6/23)	20% (4/20)	
Decompressive craniectomy	54% (23/43)	52% (12/23)	55% (11/20)	0.27
Hospital day	2 (1; 2)	2 (1; 3)	1 (1; 2)	**0.006**
Ketamine infusion	98% (210/215)	99% (98/99)	97% (112/116)	0.38
Ketamine infusion dose (mcg/kg/min)	48 (32/78)	56 (32; 78)	48 (32; 75)	0.18
Propofol infusion	48/215	27/99	21/116	0.13
Propofol bolus	12/215	7/99	5/116	0.39
Fentanyl infusion	133/215	92/99	71/116	0.89
Fentanyl bolus	29/215	13/99	16/116	0.99
Dexmedetomidine infusion	0/215	0/99	0/116	—
Dexmedetomidine bolus	0/215	0/99	0/116	—
Benzodiazepine infusion	78/215	37/99	41/116	0.54
Benzodiazepine bolus	16/215	10/99	6/116	0.2
Pentobarbital infusion	3/215	1/99	2/116	0.99
Cisatracurium infusion	36/215	19/99	17/116	0.46
Cisatracurium bolus	0/215	0/99	0/116	—
3% NaCl infusion	8/215	4/99	4/116	0.99
Hypertonic saline bolus	9/215	3/99	6/116	0.51
Mannitol bolus	4/215	3/99	1/116	0.34
ICP before ketamine bolus (mmHg)	25 (19; 30)	27 (22; 32)	24 (17; 27)	**0.001**
CPP before ketamine bolus (mmHg)	68 (58; 83)	65 (55; 74)	72 (61; 88)	**0.001**
Ketamine bolus dosage (mg)	150 (100; 200)	150 (12; 180)	140 (100; 200)	0.31

**Table 2:** Variables independently associated with reduction in ICP and increase in CPP.

Variables	*B* value	95% CI	*P* value
ICP			
ICP prior to ketamine	−0.64	−0.77 to −0.51	<0.001
Days since admission	−1.23	−2.4 to −0.42	<0.003
Patient weight (kg)	0.08	0.01 to 0.17	0.04
Decompressive craniectomy	2.79	0.16 to 5.42	0.04
CPP			
CPP prior to ketamine	−0.45	−0.55 to −0.34	<0.001
Paralytic infusion	8.71	3.58 to 13.84	0.001
Benzodiazepine bolus w/ketamine	6.99	0.27 to 13.71	0.04
Decompressive craniectomy	2.79	0.16 to 5.42	0.04

## Data Availability

The deidentified raw data supporting the conclusions of this article will be made available by the authors, without undue reservation, to any qualified researcher.

## Supplementary Materials

Supplementary Materials Univariate analysis of association between clinical variables and decreased ICP and increased CPP after ketamine bolus.
