# Supplementary material for: Ketamine Boluses Are Associated with a Reduction in Intracranial Pressure and an Increase in Cerebral Perfusion Pressure: A Retrospective Observational Study of Patients with Severe Traumatic Brain Injury
Source: Crit Care Res Pract. 2022 May 21;2022:3834165. doi: 10.1155/2022/3834165 (PMC9148235; doi:10.1155/2022/3834165)
Supplement: Supplementary Materials — Univariate analysis of association between clinical variables and decreased ICP and increased CPP after ketamine bolus. [file 3834165.f1.docx]

**Ketamine Boluses Are Associated with a Reduction in Intracranial Pressure and an Increase in Cerebral Perfusion Pressure: A Retrospective Observational Study of Patients with Severe Traumatic Brain Injury**

| **Table S1:** Univariate analysis of association between clinical variables and decreased ICP after ketamine bolus | | | |
| --- | --- | --- | --- |
| **Variables** | **Decreased ICP after Ketamine bolus** | | **P value** |
|  | **No**  **(n=63)** | **Yes**  **(n=151)** |  |
| Age [years] | 26 (20;44) | 27 (20;38) | 0.45 |
| Presenting GCS | 5 (3;7) | 5 (3;7) | 0.79 |
| EDH | 4/63 | 7/151 | 0.61 |
| SAH | 40/63 | 109/151 | 0.21 |
| SDH | 42/63 | 82/151 | 0.10 |
| Contusions | 42/63 | 93/151 | 0.48 |
| AIS | 1/63 | 1/151 | 0.52 |
| Decompressive craniectomy | 34/63 | 68/151 | 0.19 |
| Days since admission | 1 (1;2) | 2 (1;3) | 0.12 |
| Ketamine bolus dose [mg] | 160 (120;200) | 150 (100;200) | 0.31 |
| Ketamine infusion dose [mcg/kg/min] | 48 (32;80) | 50 (32;78) | 0.73 |
| Propofol infusion | 15/63 | 33/151 | 0.76 |
| Propofol bolus | 3/63 | 9/151 | 0.73 |
| Fentanyl infusion | 37/63 | 95/151 | 0.57 |
| Fentanyl bolus | 7/63 | 22/151 | 0.50 |
| Dexmedetomidine infusion | 0/63 | 0/151 | - |
| Dexmedetomidine bolus | 0/63 | 0/151 | - |
| Benzodiazepine infusion | 21/63 | 55/151 | 0.64 |
| Benzodiazepine bolus | 4/63 | 12/151 | 0.69 |
| Pentobarbital infusion | 2/63 | 1/151 | 0.16 |
| Cisatricurium infusion | 6/63 | 30/151 | 0.07 |
| Cisatricurium bolus | 0/63 | 0/151 | - |
| Hypertonic saline infusion | 3/63 | 5/151 | 0.61 |
| Hypertonic saline bolus | 4/63 | 5/151 | 0.31 |
| Mannitol bolus | 1/63 | 3/151 | 0.85 |
| ICP before ketamine bolus [mmHg] | 23 (14;27) | 26 (21;31) | <0.001 |
| CPP before ketamine bolus [mmHg] | 68 (58;82) | 68 (8;84) | 0.86 |
| GCS: Glasgow Coma Scale; EDH: Extra-Dural Hemorrhage; SAH: Sab-Arachnoid Hemorrhage; SDH: Sub-Dural Hemorrhage; AIS: Acute Ischemic Stroke; ICP: Intra-Cranial Pressure; CPP: Cerebral Perfusion Pressure | | | |

| **Table S2:** Univariate analysis of association between clinical variables and increased CPP after ketamine bolus | | | |
| --- | --- | --- | --- |
| **Variables** | **Increase CCP after Ketamine bolus** | | **P value** |
|  | **No**  **(n=90)** | **Yes**  **(n=124)** |  |
| Age [years] | 26 (20;40) | 27 (20-39) | 0.95 |
| Presenting GCS | 5 (3;7) | 5 (3;7) | 0.73 |
| EDH | 3/30 | 8/124 | 0.31 |
| SAH | 61/90 | 88/124 | 0.62 |
| SDH | 53/90 | 71/124 | 0.81 |
| Contusions | 58/90 | 77/124 | 0.72 |
| AIS | 1/90 | 1/124 | 0.82 |
| Decompressive craniectomy | 44/90 | 58/124 | 0.76 |
| Days since admission | 1 (1;2) | 2 (1;3) | 0.06 |
| Ketamine bolus dose [mg] | 130 (100;175) | 150 (120;200) | 0.04 |
| Ketamine infusion dose [mcg/kg/min] | 48 (32;72) | 56 (40;80) | 0.14 |
| Propofol infusion | 16/90 | 32/124 | 0.17 |
| Propofol bolus | 5/90 | 7/124 | 0.98 |
| Fentanyl infusion | 54/90 | 78/124 | 0.66 |
| Fentanyl bolus | 11/90 | 18/124 | 0.63 |
| Dexmedetomidine infusion | 0/90 | 0/124 | - |
| Dexmedetomidine bolus | 0/90 | 0/124 | - |
| Benzodiazepine infusion | 31/90 | 46/124 | 0.66 |
| Benzodiazepine bolus | 6/90 | 10/124 | 0.70 |
| Pentobarbital infusion | 2/90 | 1/124 | 0.38 |
| Cisatricurium infusion | 15/90 | 21/124 | 0.96 |
| Cisatricurium bolus | 0/90 | 0/124 | - |
| Hypertonic saline infusion | 3/90 | 5/124 | 0.79 |
| Hypertonic saline bolus | 6/90 | 3/124 | 0.13 |
| Mannitol bolus | 1/90 | 3/124 | 0.48 |
| ICP before ketamine bolus [mmHg] | 23 (18;27) | 26 (22;32) | 0.002 |
| CPP before ketamine bolus [mmHg] | 77 (63;91) | 64 (55;74) | <0.001 |
| GCS: Glasgow Coma Scale; EDH: Extra-Dural Hemorrhage; SAH: Sab-Arachnoid Hemorrhage; SDH: Sub-Dural Hemorrhage; AIS: Acute Ischemic Stroke; ICP: Intra-Cranial Pressure; CPP: Cerebral Perfusion Pressure | | | |
